# Reinforcement learning control method for greenhouse vegetable irrigation driven by dynamic clipping and negative incentive mechanism

**DOI:** 10.3389/fpls.2025.1632431

**Published:** 2025-11-06

**Authors:** Ruipeng Tang, Jianxun Tang, Mohamad Sofian Abu Talip, Narendra Kumar Aridas, Binghong Guan

**Affiliations:** 1 Department of Electrical Engineering, Faculty of Engineering, University of Malaya, Kuala Lumpur, Malaysia; 2 Faculty of Electronics and Electrical Engineering, Zhaoqing University, Zhaoqing, Guangdong, China; 3 Faculty of Business and Economics, University of Malaya, Kuala Lumpur, Malaysia

**Keywords:** irrigation prediction method, greenhouse vegetable irrigation, reinforcement learning algorithm, sustainable agricultural development, greenhouse vegetable production

## Abstract

Greenhouse vegetable production was a complex agricultural system influenced by multiple interrelated environmental and management factors. Its irrigation control was a critical but not singularly decisive component. Traditional irrigation methods often caused the water wastage, uneven resource utilization and limited adaptability to dynamic environmental conditions, thereby hindering the sustainable production efficiency. To address these challenges comprehensively, this study proposed an advanced irrigation control method by utilizing the enhanced reinforcement learning approach. The Enhanced Negative-incentive Proximal Policy Optimization (ENPPO) algorithm is introduced, which integrates the dynamic clipping functions and negative incentives to manage the intricacies of continuous action spaces and high-dimensional environmental states. By incorporating real-time sensor data and historical irrigation records, the ENPPO algorithm accurately predicts the optimal irrigation volumes aligned with various vegetable growth stages. Experimental results showed that ENPPO algorithm outperforms conventional methods such as PPO and TRPO in prediction accuracy, convergence efficiency and water resource utilization. It minimized both excessive and insufficient irrigation scenarios, thus promoting enhanced vegetable yield and quality while simultaneously reducing agricultural production costs. Overall, this study presented the versatile technical solution for intelligent irrigation management within greenhouse systems, highlighting its substantial potential to advance sustainable agricultural practices.

## 1 Introduction

Nowadays, most greenhouse vegetables in the northern regions of Vietnam and Thailand primarily rely on irrigation methods such as drip irrigation, sprinkler systems and soil moisture-based quantitative irrigation. Although these techniques are widely recognized as modern methods, suboptimal management and inadequate implementation often result in the water wastage and uneven resource utilization. The significant disparities in technological advancement and operational management between farms exacerbate these issues. The excessive irrigation was combined with the poor water quality characterized by high concentrations of sodium, chloride, bicarbonates and inadequate drainage, which contributes to the soil salinization and waterlogging, so the conditions negatively affect the vegetable growth and significantly reduce the overall crop quality (Chi et al., 2021; Wang et al., 2022). Additionally, the insufficient scientific knowledge among farmers regarding the optimal irrigation practices frequently leads to inappropriate water applications, which disregards the essential factors such as the specific soil characteristics, crop water requirements and local environmental conditions.

Moreover, the excessive or poorly managed irrigation, especially in areas with slopes and vulnerable soil types, can lead to severe soil erosion and water pollution. The runoff from such irrigated lands, often contaminated with residual pesticides and chemical fertilizers due to the excessive application practices, poses substantial risks to local water bodies and ecosystems (Farooq et al., 2021; Parkash et al., 2021). Considering these challenges, the current irrigation practices often fall short of effectively matching precise irrigation volumes with dynamic crop growth requirements and rapidly changing environmental conditions. To address these gaps, this study aims to develop and rigorously validate a reinforcement-learning–based irrigation control framework that (i) integrates real-time environmental sensing with crop growth state, (ii) adaptively optimizes irrigation volumes under non-stationary conditions, and (iii) quantifies gains in water-use efficiency, yield/quality, and operating cost versus conventional rule- or threshold-based methods. By leveraging online learning and adaptive decision policies, the proposed approach targets precise, context-aware irrigation that better matches dynamic crop requirements and rapidly changing environments while reducing waste and environmental externalities.

## 2 Literature review

In order to solve the above problems, some scholars had combined the intelligent irrigation technology and achieved some achievements. The simulation-based approaches have been employed to model agroecosystem interactions and optimize irrigation schedules under variable climate conditions (Tolomio and Casa, 2020). Machine learning techniques, particularly deep learning models such as long short-term memory (LSTM) networks, have been used to predict soil moisture content and irrigation needs across different soil types and crop categories (Kashyap et al., 2021). Remote sensing methods utilizing OPTRAM and satellite data have allowed for large-scale monitoring of irrigated areas (Yao et al., 2022), while sensor-driven fuzzy logic systems integrated with Arduino platforms have demonstrated effective irrigation control for crops like tomatoes and chili (Singh et al., 2022). Additional developments include edge computing-based fertigation systems (Tran et al., 2023), rainwater pipe irrigation techniques (Marimuthu et al., 2024), fuzzy irrigation models for subtropical orchards (Xie et al., 2022), and decision support systems such as IrrigaSys that calculate soil-water balance using hydrological models (Torres-Sanchez et al., 2020). Smart precision irrigation platforms with IoT communication capabilities have also emerged for optimizing irrigation in remote (Xu et al., 2019), arid regions (Benzaouia et al., 2023; Hoque et al., 2023). Moreover, most traditional irrigation control systems lack the robustness needed to operate in high-dimensional state spaces characteristic of modern agricultural systems. In complex environments where crop water needs are influenced by numerous interacting variables—such as growth stage, solar radiation, wind, evapotranspiration, and drainage—existing models are either too simplistic or computationally inefficient. Algorithms such as PPO (Proximal Policy Optimization) have been applied in control tasks but suffer from performance degradation in the presence of fixed clipping ranges and limited response to negative incentives, making them less effective in dynamic, non-stationary environments (Ibrahim et al., 2024).

These limitations significantly hinder accurate and timely irrigation decision-making under real-world greenhouse or open-field conditions. Against this backdrop, the motivation of this study is to bridge these specific performance gaps by improving prediction accuracy, adaptability to complex environmental changes, and computational efficiency in real-time irrigation control. Although reinforcement learning (RL) and deep learning methods have gained traction in recent years, their application in greenhouse vegetable irrigation remains constrained by weak generalizability, slow convergence, and poor adaptability to continuous action spaces. The main research gaps identified are as follows:

1. Limited multidimensional adaptability: Most existing models, especially fuzzy systems and static optimization frameworks, lack mechanisms to dynamically respond to the multi-factor environmental conditions across crop growth stages.
2. Algorithmic rigidity: Standard RL approaches like PPO show the promising convergence under ideal conditions but fail to dynamically adjust their update mechanisms (e.g., clipping range) or penalize ineffective decisions, which reduces robustness in complex agricultural settings.
3. Lack of general-purpose, crop-sensitive irrigation prediction models: It remains an absence of intelligent irrigation algorithms that combine high prediction accuracy, rapid convergence and adaptability across different vegetables and growth phases.

In response to these gaps, this study proposed a novel irrigation control method based on the enhanced negative-incentive proximal policy optimization (ENPPO) algorithm. The primary contributions include:

1. Problem definition and scope: The study targets the accurate prediction of irrigation volume for greenhouse vegetables (including Chinese pakchoi, Shanghai greens, and Komatsuna) at various growth stages by integrating a comprehensive set of environmental factors in real-time.
2. Algorithmic innovation: The ENPPO model extended the standard PPO framework by incorporating a dynamic clipping mechanism and negative incentive modulation, enabling more adaptive and penalizing learning in high-dimensional, non-stationary environments.
3. Empirical validation: Experimental trials conducted in semi-enclosed greenhouses in Vietnam’s Tam Dao District demonstrate that the ENPPO algorithm achieved superior water-use efficiency, faster convergence, and more stable yield outcomes compared to benchmark algorithms (PPO and TRPO).

In summary, this study applied an improved reinforcement learning framework to greenhouse vegetable irrigation control for the first time. It directly addressed the shortcomings of prior work by delivering the model that excels in prediction accuracy, environmental adaptability and computational robustness—key factors for advancing intelligent irrigation in sustainable agriculture.

## 3 Materials and methods

### 3.1 Overall research process

This study covers the complete chain from data acquisition to model training and control evaluation. First, raw data is acquired through multimodal soil and environmental sensors and meteorological stations, and traceability calibration is completed before deployment, and weekly reviews are conducted during operation. Subsequently, the data is subjected to quality control, missing value and outlier processing, threshold screening is performed based on the detection limit and quantification limit of the sensor, and data from different sources are uniformly time-aligned. On this basis, feature engineering and state construction are carried out, each channel is masked and uncertainty marked, and data batches that can be directly used for model training and inference are generated. The improved reinforcement learning algorithm is trained and validated on this dataset, including dataset partitioning, hyperparameter management and policy output. The trained strategy is deployed online in the greenhouse control system and supplemented by safety constraint mechanisms such as execution frequency control, threshold protection and fallback strategy. During operation, the system monitors and records operation logs in real time and performs anomaly detection. Finally, the performance of different methods is evaluated using indicators such as water-saving efficiency, drainage events, salinity drift, soil moisture error, and the number of steps required to reach the threshold. If the evaluation results are unsatisfactory, the feature and parameter layers are returned for iterative updates to ensure the traceability and reproducibility of the research process.

### 3.2 Experimental setup

**Figure 1:**
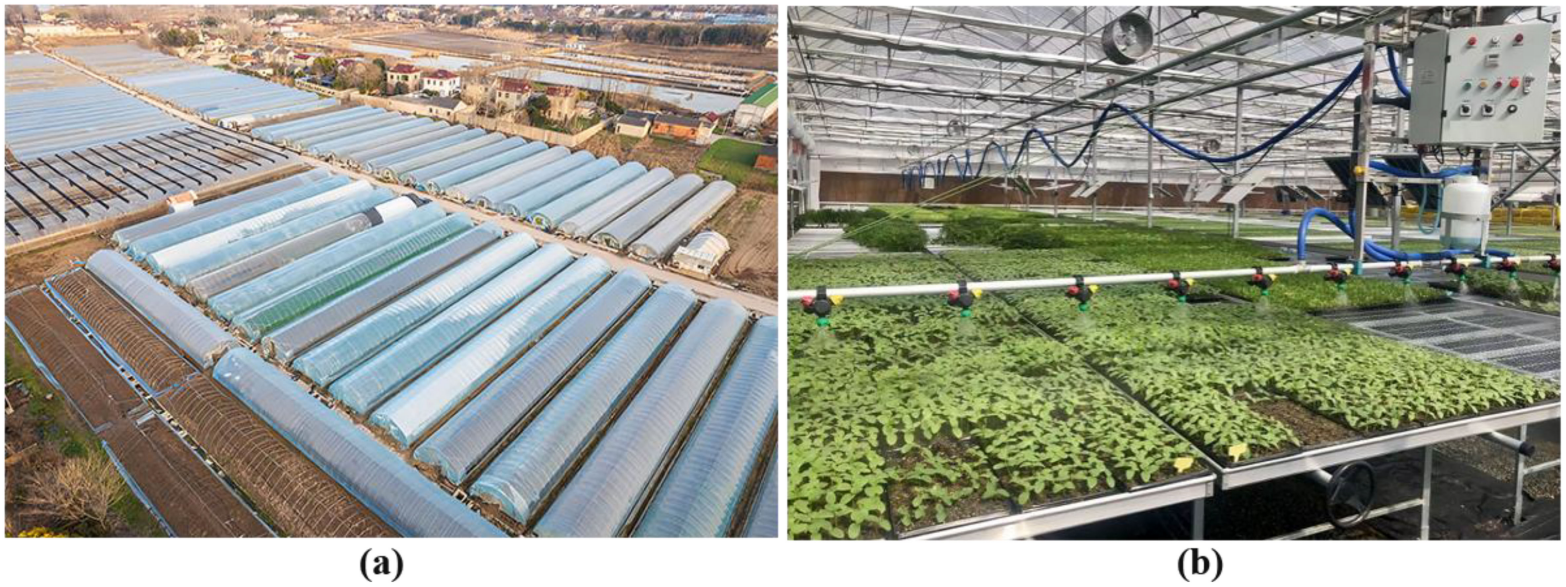
The greenhouse vegetable experimental base: **(A)** aerial photo; **(B)** internal water and fertilizer irrigation equipment.

This study selected the growth data from VinEco vegetable planting base in Tam Dao District, Vinh Phuc Province, Vietnam. This study focused on three commonly cultivated greenhouse leafy vegetables—Chinese Pakchoi (Brassica rapa subsp. chinensis), Shanghai green (a type of Brassica rapa var. communis) and Komatsuna (Brassica rapa var. perviridis). The irrigation control strategy was developed and tested across three physiologically distinct growth stages: seedling (emergence to 4–6 true leaves), vegetative growth (leaf expansion and biomass accumulation) and maturity (pre-harvest phase). By analyzing the irrigation demands and environmental responses across these stages, the study aims to reflect the dynamic water requirements of leafy vegetables under variable greenhouse conditions. The crops were cultivated at a commercial production density with 15 cm plant spacing and 20 cm row spacing across a 500 m² experimental site. This experimental greenhouse was a semi-enclosed structure, and some areas were affected by external climate conditions (such as precipitation and temperature changes). The intelligent water-fertilizer integrated irrigation equipment was used in the greenhouse, which can adjust the amount of irrigation and fertilization according to demand. The greenhouse area was equipped with the environmental control system that adjusted the temperature and humidity to meet the growth needs of vegetables. The outdoor planting area was equipped with a drip irrigation and sprinkler irrigation system for irrigation management of vegetables in different growth stages. In order to improve the economic benefits, most greenhouses used the integrated water and fertilizer equipment, so the farm managers can decide whether to irrigate or fertilize according to the demand. **Figure 1** shows the greenhouse vegetable experimental base: (A) aerial photo; (B) internal water and fertilizer irrigation equipment.

### 3.3 Data collection

**Figure 2:**
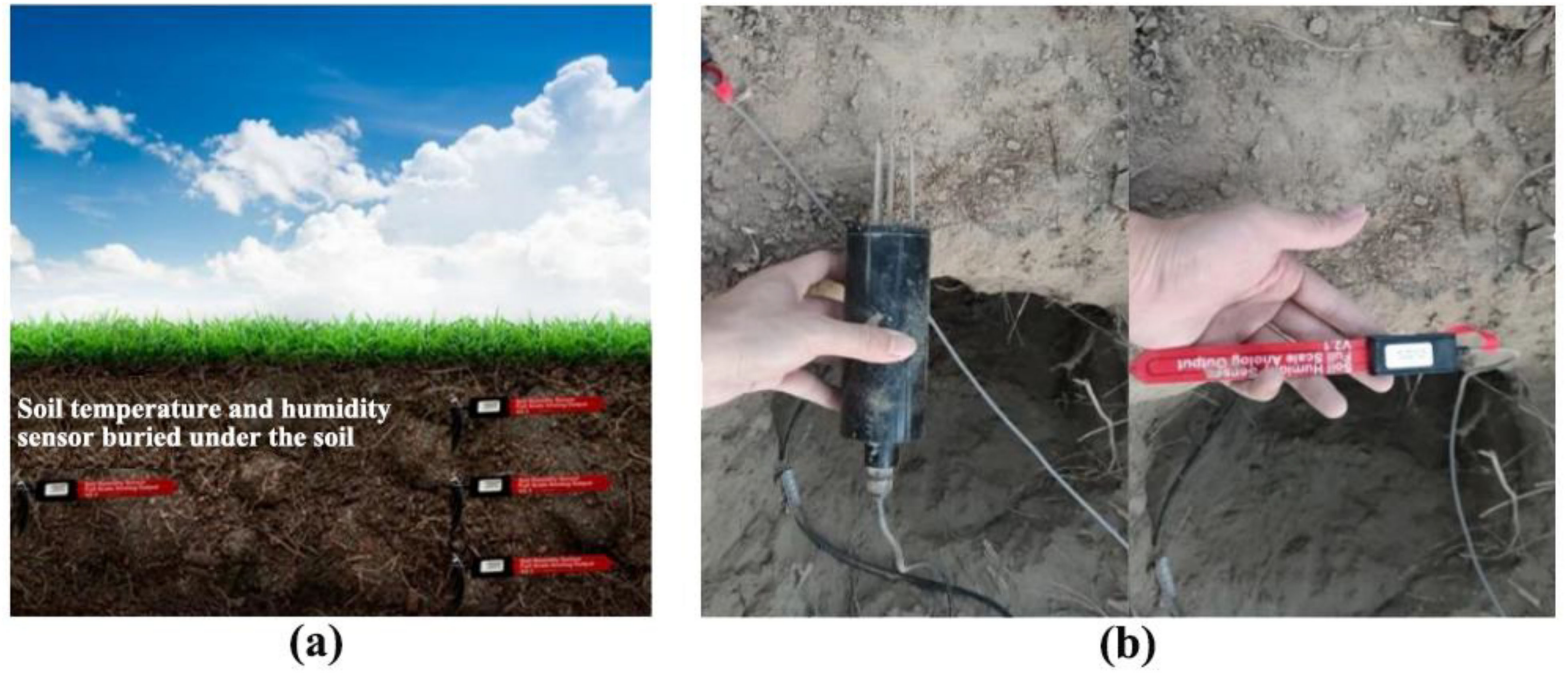
The soil temperature and moisture sensor placement during the growth stage in the field: **(A)** The soil sensor placement site; **(B)** The actual soil temperature and moisture sensor.

This study used the Rk510–01 agricultural soil moisture sensor produced by Hunan Ruika Electronic Technology Co., Ltd., with an error range of ±0.1°C and ±0.5%. It also used the FT-WQX7 six-element micro-meteorological sensor produced by Shandong Jingdao Optoelectronic Technology Co., Ltd. to monitor environmental parameters such as humidity, wind speed, wind direction, atmospheric pressure and optical rainfall, with an error range of ±1%. These sensors were calibrated before the experiment to ensure the accuracy and reliability of the data. This study collected data through soil temperature and humidity sensors, environmental climate sensors and water-fertilizer integrated equipment. The period was from January 1, 2023, to June 30, 2023, a total of 180 days, which includes about 30,000 valid data records. Under the irrigation of water-fertilizer equipment, the soil layer with the highest moisture content of the above vegetables appears at 70~100 mm, so the soil moisture sensor was set to be buried 90mm from the ground surface, and the soil temperature sensor was buried 70mm from the ground surface. The soil temperature and moisture sensor placement during the growth stage in the field is shown in **Figure 2**. The soil moisture sensors (Rk510-01) and climate sensors (FT-WQX7) provided data at 10-minute intervals and were resampled to match the 30-minute reinforcement learning time steps. The sensors were calibrated weekly by using gravimetric soil sampling and standardized environmental chambers to ensure the measurement error (<5%). The planting density data (15 cm plant spacing, 20 cm row spacing) was encoded into the state space vector via crop water use coefficients during the action-value update step.

To strengthen reproducibility, this study explicitly define limits of detection (LOD) and limits of quantification (LOQ) for all environmental and soil sensors and align calibration and preprocessing with these thresholds. LOD/LOQ are established following standard analytical practice: short-term noise under blank or static conditions is characterized and the common “three-sigma” and “ten-sigma” criteria are applied, combined with manufacturer resolution and zero-drift tests; for quantities without a strict blank (e.g., wind speed, irradiance), thresholds are inferred from zero-drift under steady conditions and the minimal resolution. All sensors undergo traceable pre-deployment calibration and weekly re-verification: soil volumetric water content is checked by gravimetric oven-drying, electrical conductivity by standards, temperature/humidity/pressure in an environmental chamber, wind speed/direction in still or constant-speed tunnels, and irradiance with a standard light source; noise estimates and LOD/LOQ values are updated accordingly. Preprocessing follows unified rules: readings below LOD are treated as non-detects, masked out of model inputs, and excluded from statistical tests; readings between LOD and LOQ are retained with high-uncertainty flags, down-weighted in training losses/rewards and in evaluation statistics with explicit sensitivity tagging; readings at or above LOQ are considered quantifiable and are fully used for modeling and performance assessment. For transparency, we provide a consolidated table of sensor ranges, resolutions, and the corresponding LOD/LOQ, together with weekly calibration logs and threshold-update notes, enabling independent researchers to reproduce state construction and statistical conclusions under the same gates.

### 3.4 Irrigation control modeling based on reinforcement learning

The reinforcement learning environment built the virtual interactive simulation system that enables the intelligent agent to continuously optimize the decision-making strategy to achieve specific goals through the environmental interaction. In this study, the construction of reinforcement learning environment focused on solving the greenhouse vegetable irrigation control problem. It simulated the dynamic impact of different environmental variables on irrigation decisions through algorithms to achieve the precise irrigation. It enabled the agent to interact with the environment through a series of observations, actions and feedback values so that the optimal strategy can be found in the unknown environment to maximize the cumulative reward value (Zhao et al., 2023). This study constructed a reinforcement learning environment based on the growth characteristics of greenhouse vegetables and environmental influencing factors, as expressed in Equation 1:

(1) L={A, B, C, O}

In Equation 1, A represents the space state, B represents the space action, C represents the transition probability, O represents the reward function.  L represents that the state space refers to the decision-making environment variables. In the irrigation system, state space variables include growth stage, evapotranspiration, soil moisture content, upper soil water limit, lower soil water limit, vegetable water absorption, vegetable drainage, light intensity and so on, as shown in Equation 2:

(2) $$P_{t}=\{p_{stage},p_{trans},p_{water},p_{w\_max},p_{w\_min},p_{abso},p_{disp},p_{light}\}$$

In Equation 2, $P_{t}$ represents the state quantity of vegetable irrigation environment in time, $p_{stage}$ represents the growth stage, $p_{trans}$ represents the evapotranspiration of vegetables, $p_{water}$ represents the soil moisture content (soil available water), $p_{w\_max}$ and $p_{w\_min}$ represents that the upper and lower limits of soil water, $p_{abso}$ represents the amount of water absorbed by vegetables, $p_{disp}$ represents the drainage volume of vegetable growing areas, $p_{light}$ represents the light intensity value collected by the sensor, which is monitored by the light sensor and updated in real time to improve the responsiveness of the reinforcement learning environment to dynamic environmental changes. τ represents the amount of irrigation of action spaces. In order to satisfy the rationality and continuity of the irrigation amount during the irrigation process, this study sets four action quantities in the action space, as shown in Equation 3:

(3) $$A_{t}=\{\begin{array}{ll} 0 & sign=1\\ 0.25p_{need} & sign=2\\ 0.5p_{need} & sign=3\\ 0.75p_{need} & sign=4 \end{array}$$

In Equation 3, $A_{t}$ represents the time t in the amount of vegetable irrigation, $p_{need}$ represents the water requirement of vegetables,  sign represents the irrigation action sign. According to the vegetable environmental variables and irrigation amount, the water content at the next moment is shown as Equations 4, 5:

(4) $$B_{t}=B_{t-1}+A_{t}-p_{abso}-\;p_{trans}(p_{light})$$

In Equation 4 $,\;B_{t}$ represents the soil moisture content (soil available water) at time t-1. $p_{trans}(p_{light})\;\;$represents the light intensity $p_{light}$ is introduced into $p_{trans}$, which affects the evapotranspiration of vegetable. Under the high light intensity ( $p_{light}$), the evapotranspiration $p_{trans}$ increases; under low light intensity (low $p_{light}$), the evapotranspiration $p_{trans}$ decreases. Among them, $p_{trans}(p_{light})=\tau_{1}\times p_{basic-trans}+\;\tau_{2}\times p_{light}$, $\tau_{1}$ and $\tau_{2}$ represents the empirical coefficients, $p_{basic-trans}$ represents the basic evaporation. The feedback function is based on the changes of wheat field irrigation state variables. In this study, if the vegetable yield increases, the irrigation amount becomes relatively less and the irrigation income becomes larger, the current irrigation timing will be increased. If the yield decreases, the irrigation volume is large, and the irrigation income becomes smaller, so the feedback value is set to a negative value. When the soil moisture content exceeds the upper and lower limits of the optimal soil moisture content, the feedback value is set to be negative (Zhang et al., 2021). The feedback function is shown as Equations 5, 6:

(5) $$W=\sum_{t=1}^{T_{max}}{\text{W}}_{p,t}\times {\text{W}}_{p}+\;H\times Y_{price}\;-\sum_{t=1}^{T_{max}}B_{t}\times Y_{water}$$

(6) $$W_{t}=\{\begin{array}{ll} -12 & \;\;\;\;\;\;\;p_{t}<p_{min},p>p_{max}\\ 0 & p_{min}\leq p_{t}\leq p_{max},A_{t}=1\\ 6 & p_{min}\leq p_{t}\leq p_{max},A_{t}=2\\ 12 & p_{min}\leq p_{t}\leq p_{max},A_{t}=3 \end{array}$$

In Equations 5, 6, W represents the feedback function value, ${\text{ W}}_{p,t}$ represents the soil moisture content feedback value at time t, $T_{max}$ represents the maximum maturity time of vegetables, H represents the vegetable yield, $Y_{price}$ represents the vegetable price, $Y_{water}$ represents the price of irrigation water. In order to evaluate the irrigation strategy, the action value function U represents the cumulative expected reward value, σ represents the irrigation strategy. Under the input state j selection action ucondition, the action value function U is shown as Equation 7:

(7) $$U_{\text{j}}(\text{m},\text{n})=L_{\text{j}}\{\sum_{t=0}^{+\infty }w_{i}\times w_{i+t}❘m_{t}=m,n_{t}=n❘\}$$

In Equation 7, w represents that the discount factor is 0.2. The discount factor measures the importance of future rewards and is usually between 0 and 1. (a,g)A joint variable representing state and action. The optimal action-value function Gvalue and optimal strategy are expressed in Equations 8, 9 as follows:

(8) $$\bar{\text{U}}(\text{m},\text{n})={\text{max}}_{\text{j}}\{U_{\text{j}}(\text{m},\text{n})\}$$

(9) $$\bar{\text{V}}(\text{m},\text{n})={\text{argmax}}_{\text{n}\in \text{N}}\{U_{\text{j}}(\text{m},\text{n})\}$$

In Equations 8, 9, $\bar{\text{U}}(\text{m},\text{n})$ represents that among all possible irrigation strategies, which select the strategy that maximizes the action value function; ${\text{max}}_{\text{j}}\{U_{\text{j}}(\text{m},\text{n})\}$ represents maximization of all possible strategies. $\bar{\text{V}}(\text{m},\text{n})$ represents the optimal policy that maximizes the action-value function. ${\text{argmax}}_{\text{n}\in \text{N}}\{U_{\text{j}}(\text{m},\text{n})\}$ represents maximizing all possible actions and finding the action that maximizes the action value function.

**Figure 3:**
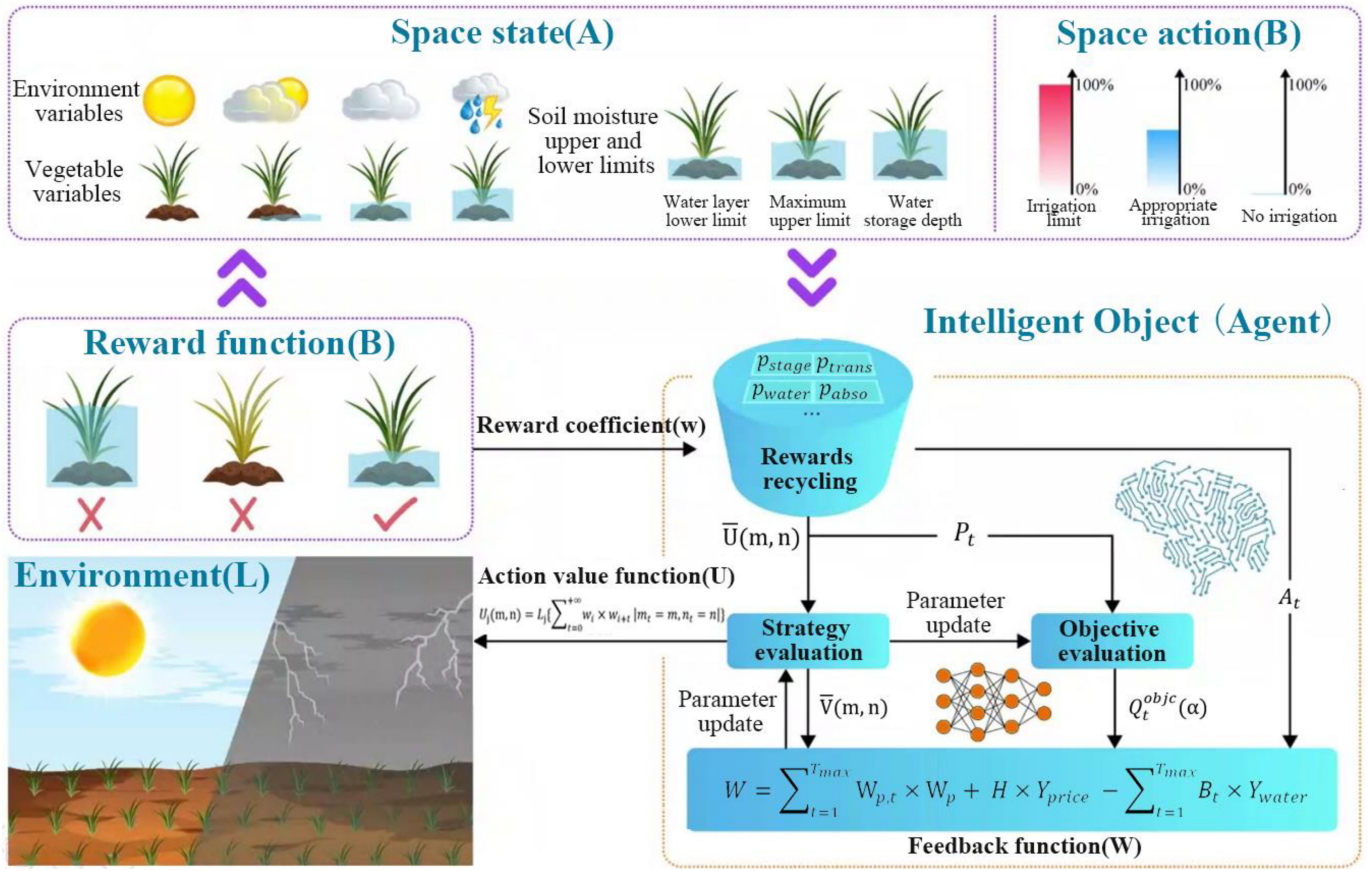
The reinforcement learning environment process for greenhouse vegetables.

**Figure 3** shows the reinforcement learning environment process for greenhouse vegetables. The reward shaping couples penalties to agronomic risk indicators: deviations from target soil-moisture bands incur proportional costs; threshold crossings in drainage/ponding indicators trigger stepped penalties to deter waterlogging; and rising soil EC/Na^+^ proxies induce incremental costs to discourage salinity build-up. These terms operationalize the negative incentive as a policy-consistent instrument for water saving and root-zone protection. Why standard reward functions are insufficient and how our mechanisms offer a superior solution. In greenhouse irrigation, conventional rewards are typically: (i) sparse and delayed, giving feedback only at episode ends or when thresholds are crossed, which weakens step-wise guidance and slows learning; (ii) stage-agnostic with fixed weights, so signals tuned for the seedling phase mislead actions during vegetative or maturity phases under non-stationary weather; and (iii) risk-blind, omitting explicit costs for agronomic hazards such as waterlogging, excessive drainage, and salinity build-up. These limitations invite reward hacking (short-term gains via over-irrigation), produce unstable policy updates, and hinder generalization across crops and growth stages. To address this, this study designs a stage-aware, risk-coupled reward and pair it with two complementary mechanisms: a negative-incentive that actively discourages actions increasing agronomic risk even when they appear profitable in the short run, and state-sensitive dynamic clipping that adapts update stringency to environmental volatility. Together, they deliver denser and context-relevant feedback, suppress unsafe exploration, stabilize optimization in continuous action spaces, and consistently achieve faster convergence, tighter moisture control, fewer drainage events, and better cross-stage robustness than standard reward functions.

### 3.5 Negative incentive dynamic PPO algorithm

The Proximal Policy Optimization (PPO) is an algorithm used to solve reinforcement learning policy optimization problems, especially problems such as continuous action space and high-dimensional state space (Gu et al., 2021). The PPO goal maintains the relative proximity between the new and old policies by introducing constraints when updating the policy to ensure stable and safe policy updates. Its optimization strategy maximizes the accumulated positive feedback value, and its objective function is expressed in Equation 10.

(10) $$Q_{t}^{objc}(\text{α})=\text{F}[\text{min}(\text{x}(\text{α})O_{t},\text{clip}(\text{x}(\text{α}),1-\text{α},1+\text{α})\times Z_{t})]$$

In Equation 10, $Q_{t}^{objc}(\text{α})$ represents the objective function, x(α) represents the output of the policy network, α represents the parameters of the policy network; $Z_{t}$ is the advantage function, represents the advantage of the current state action; F[] represents some kind of aggregation or expectation operator; min() represents the ratio of the current strategy and the old strategy. It also uses a function called “clipping” to ensure relatively small steps in policy updates by limiting the ratio of the new policy to the old policy, which is shown in Equation 11:

(11) $$clip(\text{α})=\{\begin{array}{cc} \left(1-\beta \right) & x_{t}(\text{α})\leq 1-\beta <0\\ \left(1+\beta \right) & x_{t}(\text{α})\geq 1-\beta >0\\ x_{t}(\text{α}) & otherwise \end{array}$$

In Equation 11, clip(α) represents the shear function that limits parameters, $x_{t}(\text{α})$ represents the certain range. This clipping function prevents policy updates from being too large, which keeps the steps relatively small and avoids policy divergences. In order to enable the PPO algorithm to limit the probability ratio, this study constructs a new clip function by reducing the influence of incentives, as shown in Equation 12:

(12) $$clip(x(\text{α}),1-\beta ,1+\beta )=\{\begin{array}{cc} \left(1-\beta )+\gamma [\text{tanh}(1-\beta )-x(\text{α})\right] & x_{t}(\text{α})\leq 1-\beta \\ \left(1+\beta )+\gamma [\text{tanh}(1+\beta )-x(\text{α})\right] & x_{t}(\text{α})\geq 1-\beta \\ x_{t}(\text{α}) & otherwise \end{array}$$

In Equation 12, tanh represents the hyperbolic tangent function. γ>0 represents the intensity factor, which controls the specific size of negative excitation. The gray circle on each plot represents the starting point of the optimization ( $x_{t}(\text{α})=1$). When $x_{t}(\text{α})$ runs out the clipping range, $Q_{t}^{tanh}(\text{α})\;$represents that the slope is reversed, $Q_{t}^{objc}(\text{α})$ represents that the slope is 0. The new clip function prevents the probability ratio from being overly stretched compared to the original clipping function (Corecco et al., 2023).

**Figure 4:**
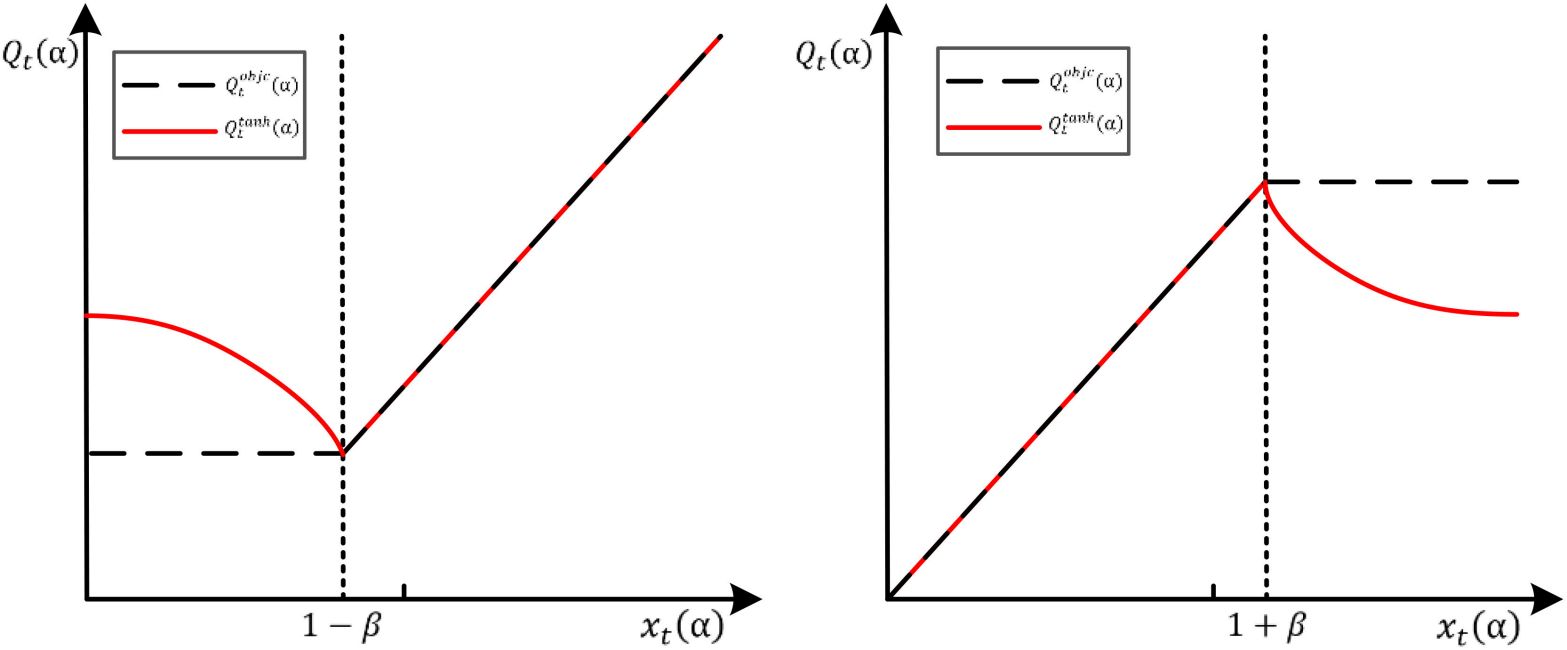
The objective function when $Z_{t}>0$ and $Z_{t}<0$.

**Figure 4** shows the objective function when $Z_{t}>0$ and $Z_{t}<0$.

However, the fixed clipping interval design ignores the degree of difference between states, which have different states of the agent correspond to different actions. It has different feedback obtained values, which restricts the PPO algorithm’s learning efficiency and convergence accuracy. In order to improve the learning efficiency and convergence accuracy of the PPO algorithm and obtain the dynamic interval limit of PPO, this study starts from KL divergence. It improves the objective function as shown in Equation 13:

(13) $$Q_{t}^{objc}(\text{α})=\{\begin{array}{cc} (1-\beta )+\gamma [\text{tanh}(1-\beta )-x(\text{α})]Z_{t} & x_{t}(\text{α})\leq 1-\beta \;and\;R_{t}<0\\ (1+\beta )+\gamma [\text{tanh}(1+\beta )-x(\text{α})]Z_{t} & x_{t}(\text{α})\geq 1-\beta \;and\;R_{t}>0\\ x_{t}(\text{α})R_{t} & otherwise \end{array}$$

**Figure 5:**
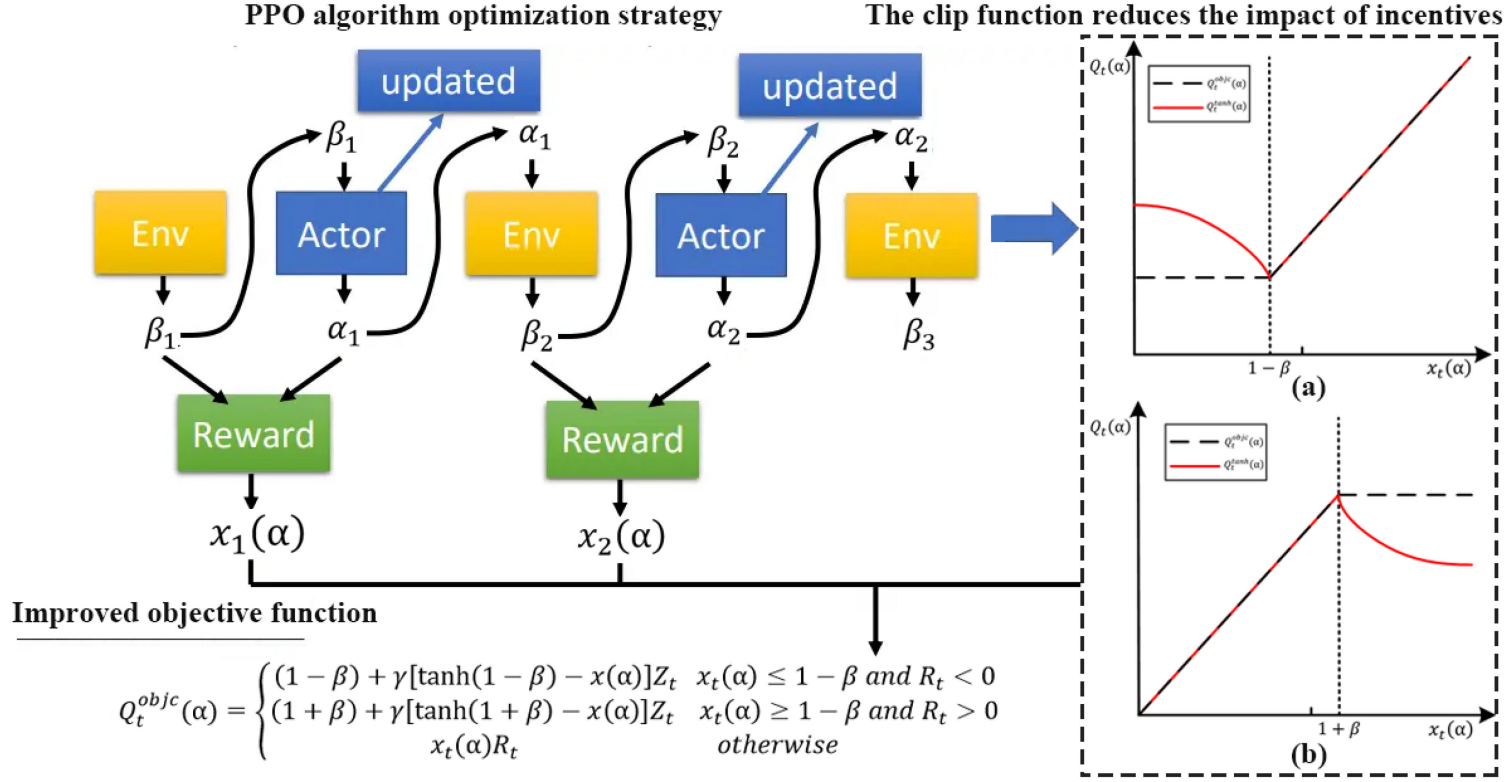
The process of optimizing reinforcement learning strategy by using negative incentive dynamic PPO algorithm.

In Equation 13, $Q_{t}^{objc}(\text{α})$ represents the improved objective function and the optimization goal at time step t. β represents the base value;  γ represents the positive number, representing the intensity factor, which controls the specific size of negative excitation. tanh(1+β)−x(α) represents the adjustment term that gradually reduces the excitation through the hyperbolic tangent function. $R_{t}$ represents the advantages of the current state action. This improved objective function takes into account the differences between states. It sets different actions according to different states to improve the learning efficiency and convergence accuracy of the PPO algorithm (Engstrom et al., 2020). **Figure 5** shows the process of optimizing reinforcement learning strategy by using negative incentive dynamic PPO algorithm

However, the fixed clipping interval design in the original PPO (as shown in Equation 11) neglects the variation in state-action relevance across different crop growth stages and environmental dynamics, such as evapotranspiration, solar radiation, and soil drainage. To address this limitation, the ENPPO algorithm introduces the state-sensitive dynamic clipping function and negative incentive term. These innovations are formalized to enhance policy adaptation in high-dimensional, non-stationary greenhouse irrigation environments. Specifically, the static clipping parameter ϵ is replaced by a state-dependent function based on the KL divergence between the updated and previous policies:

(14) $$\epsilon_{t}=\lambda \tanh(\beta \times D_{KL}(\pi_{\theta old}(.❘s_{t})❘❘\pi_{\theta }(.❘s_{t})))$$

In Equation 14, λ represents the clipping intensity factor, β represents the scaling coefficient, $D_{KL}$ represents the Kullback-Leibler divergence measuring policy change under state, $\pi_{\theta }$ and $\pi_{\theta old}$represents the current and previous policy networks. This function allowed the algorithm to adaptively control the policy update magnitude depending on environmental volatility and the agent’s response behavior. In addition, to penalize ineffective or excessive irrigation strategies, the negative incentive term ${\text{δ}}_{t}$ is incorporated. It is constructed using the current policy advantage and the environmental feedback function $W_{t}$ (from Equation 6), which encodes penalties for over-irrigation, yield loss, and deviations from optimal soil moisture:

(15) $${\text{δ}}_{t}=\alpha \tanh(-{\text{γ}}_{t}(\theta )\times A_{t})+\;\gamma \;\times W_{t}$$

In Equation 15, α represents the penalty coefficient for adverse advantage values, γ represents the environmental penalty from moisture deviation and yield loss, ${\text{γ}}_{t}(\theta )$ represents the probability ratio from Equation 10, $A_{t}$ represents the advantage function, $W_{t}$ is computed as in Equation 6 using current soil moisture, yield, and irrigation cost. The negative incentive term effectively dampens updates when strategies lead to over-irrigation or poor economic performance, encouraging the model to explore more optimal action sequences under dynamic crop conditions. Combining the above mechanisms, the revised ENPPO objective function is defined as:

(16) $${\text{L}}_{ENPPO}(\theta )=\alpha \tanh(-{\text{γ}}_{t}(\theta )\times A_{t})+\;\gamma \;\times W_{t}$$

In Equation 16, it integrates the policy stability through dynamic clipping ( $\epsilon_{t}$), the penalty sensitivity via negative incentive ( ${\text{δ}}_{t}$), the environmental context via ( $W_{t}$) and the light/evapotranspiration signals from the sensor system. As demonstrated in the experimental results (Section 4, Figures 10 and 11), this enhanced structure significantly improves the convergence rate, irrigation precision, and policy robustness compared to PPO and TRPO, confirming its theoretical and practical effectiveness in complex greenhouse irrigation control. So in order to provide greater specificity of the novel mechanisms, we emphasize that the dynamic clipping function directly addresses the inefficiency of PPO under fluctuating soil moisture and evapotranspiration, while the negative incentive mechanism explicitly penalizes excessive drainage and salinity accumulation. Experimental ablation results show that dynamic clipping alone shortened convergence steps by 22.52%, while negative incentive reduced drainage events by 32.99%. When combined, ENPPO achieved an overall 36.69% reduction in soil moisture error and a 22.15% improvement in WUE compared to baseline PPO, highlighting the complementary nature of the two mechanisms.

### 3.6 Improved reinforcement learning algorithm

Although traditional reinforcement learning algorithms can solve discrete control decision-making problems, they face the problem of slow speed. Considering that the irrigation model proposed in this study is a continuity model, the convergence optimization efficiency of the existing deep reinforcement learning model is not high (Cui et al., 2023). This study used the PPO algorithm to solve the irrigation control optimization problem and proposed an enhanced reinforcement learning algorithm (ENPPO). The BPNN used in the ENPPO model consisted of an input layer of 14 environmental variables, two hidden layers with 64 and 128 neurons respectively and an output layer for irrigation decision value. ReLU activation was used, and the Adam optimizer updated network weights every 20 steps. The batch size was 64, and the total number of iterations per stage was set to 1500. All state-action-reward transitions were stored in an experience replay buffer of size 20,000 and resampled randomly during training. According to the PPO algorithm, the action value function $G_{\text{σ}}(\text{a},\text{g})$is updated as shown in Equation 17:

(17) $$U({\text{m}}_{t},{\text{n}}_{t})=U({\text{m}}_{t},{\text{n}}_{t})+n[x_{t}+\alpha max_{n_{t+1}}U({\text{m}}_{t+1},{\text{n}}_{t+1})-U({\text{m}}_{t},{\text{n}}_{t})]$$

In Equation 17, α represents the learning rate, which is set to 0.001, 0.003, 0.003 and 0.002 in the germination, seedling, growth and maturity stages. In order to handle the continuous state space, this study uses BPNN (back-propagation neural network) (Duan et al., 2023) to replace the approximate function. Vegetable growth Influencing factors such as state, water absorption, evapotranspiration, crop drainage, and soil available water are continuously trained by minimizing the loss function. The loss function is shown in Equation 18:

(18) $$U({\text{n}}_{k})=F[{\left(x+\varphi max_{{\text{n}}_{t+1}}U({\text{m}}_{t+1},{\text{n}}_{t+1};\bar{{\text{n}}_{k}})-U({\text{m}}_{t},{\text{n}}_{t};{\text{n}}_{k})\right)}^{2}]$$

In Equation 18, $x+\varphi max_{{\text{n}}_{t+1}}U({\text{m}}_{t+1},{\text{n}}_{t+1};\bar{{\text{n}}_{k}})$ represents the target of the k- th iteration, $\bar{{\text{n}}_{k}}$ represents the parameters of the calculation target network, ${\text{n}}_{k}$ represents the parameters of the action network of the k-th iteration. The parameter companion of each step state-action network are updated by the parameters of the target network. The action value function is adjusted by updating the parameters of the state-action network, which is shown in Equations 19, 20:

(19) $${\text{n}}_{k+1}={\text{n}}_{k}+\omega S_{{\text{n}}_{k}}D_{k}({\text{n}}_{k})$$

(20) $$\omega S_{{\text{n}}_{k}}D_{k}({\text{n}}_{k})\;=F[(x+\varphi max_{{\text{n}}_{t+1}}U({\text{m}}_{t+1},{\text{n}}_{t+1};\bar{{\text{n}}_{k}})-U({\text{m}}_{t},{\text{n}}_{t};{\text{n}}_{k}))\times S_{{\text{n}}_{k}}U({\text{m}}_{t},{\text{n}}_{t};{\text{n}}_{k})]$$

In Equation 19, ${\text{n}}_{k+1}$ and ${\text{n}}_{k}$ represents the parameters of the state-action network of the k+1 and k iterations;  ω represents the learning rate, which controls the update step of the parameters; $\;S_{{\text{n}}_{k}}D_{k}({\text{n}}_{k})$ represents the direction in which the parameters ${\text{n}}_{k}$ are updated. In Equation 20, φ represents the parameter, $max_{{\text{n}}_{t+1}}U({\text{m}}_{t+1},{\text{n}}_{t+1};\bar{{\text{n}}_{k}})$ represents the current state-action value function under the action network parameters ${\text{n}}_{k}$; $S_{{\text{n}}_{k}}U({\text{m}}_{t},{\text{n}}_{t};{\text{n}}_{k})$ represents ${\text{n}}_{k}$ is the gradient of the action value function with respect to the parameters. It combines the reinforcement learning model and PPO algorithm.

**Figure 6:**
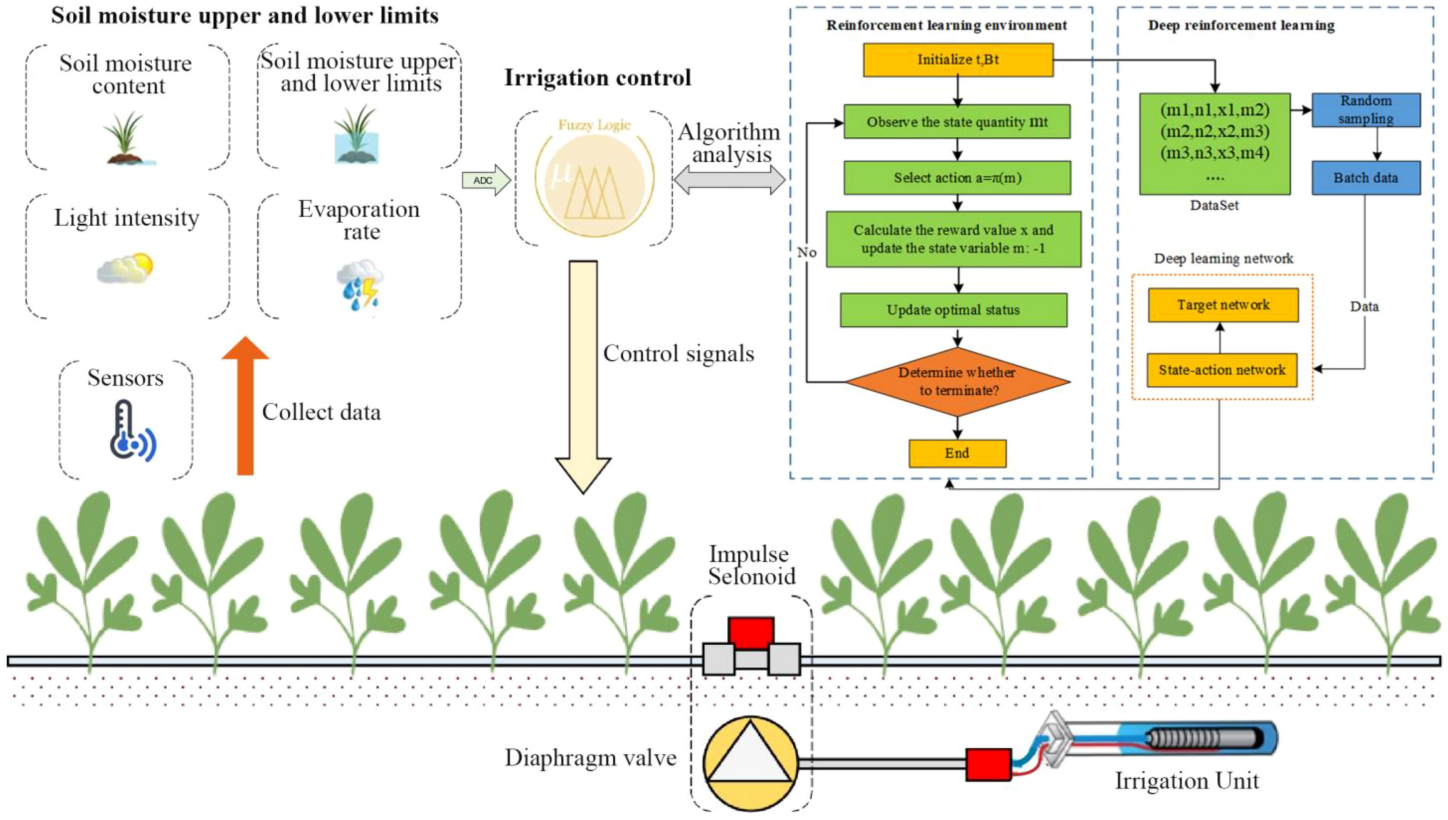
The prediction process of vegetable irrigation amount based on the ENPPO algorithm.

**Figure 6** shows the prediction process of vegetable irrigation amount based on the ENPPO algorithm. The first step is to initialize the parameters, which includes the initialization reward discount coefficient, experience pool size, target network update steps, number of iterations, single maximum number of steps, number of randomly selected samples, etc. ${\text{m}}_{1}$, ${\text{n}}_{1}$, ${\text{x}}_{1}$, ${\text{m}}_{2}$ represent the water absorption, evapotranspiration, drainage and effective soil moisture of the plants in the experiment. All parameters are derived from the experimental data collected by sensors. The second step is to build an irrigation prediction environment to initialize state variables, which includes randomly selecting the amount of action space, calculating reward values, updating the amount of state space, etc. The third step is to randomly sample training samples from the data set to update the state-action network. The fourth step uses the state-action network parameters to update the target network and inputs the current action amount to update the environment state to calculate the reward value. Finally, it is judged that the maximum number of iterations has been reached. If reached, the optimal action sequence is output; otherwise, continue to step 3. All simulation models, data generation code, sensor parameters, and hyperparameter configurations used in this study are available upon request. The greenhouse simulation system was implemented in Python 3.9 by using Numpy, Scikit-learn and PyTorch 1.13. The RL environment was built on OpenAI Gym with custom wrappers for greenhouse irrigation tasks. Time steps were fixed at 30 minutes, with one episode covering a 180-day crop cycle. The random seeds were fixed at 42 for reproducibility, and external weather data were sampled from three-year averages at the Tam Dao station (NOAA).

## 4 Experimental result

In order to verify the performance of the ENPPO algorithm, it was compared with the TRPO (Trust Region Policy Optimization) (Cen et al., 2022) and PPO (Proximal Policy Optimization) (Schulman et al., 2017) algorithm. The overall goal of this experiments is to verify the effectiveness of the ENPPO algorithm by analyzing the performance of irrigation control. The experimental results are as follows:

### 4.1 Algorithm stability

**Figure 7:**
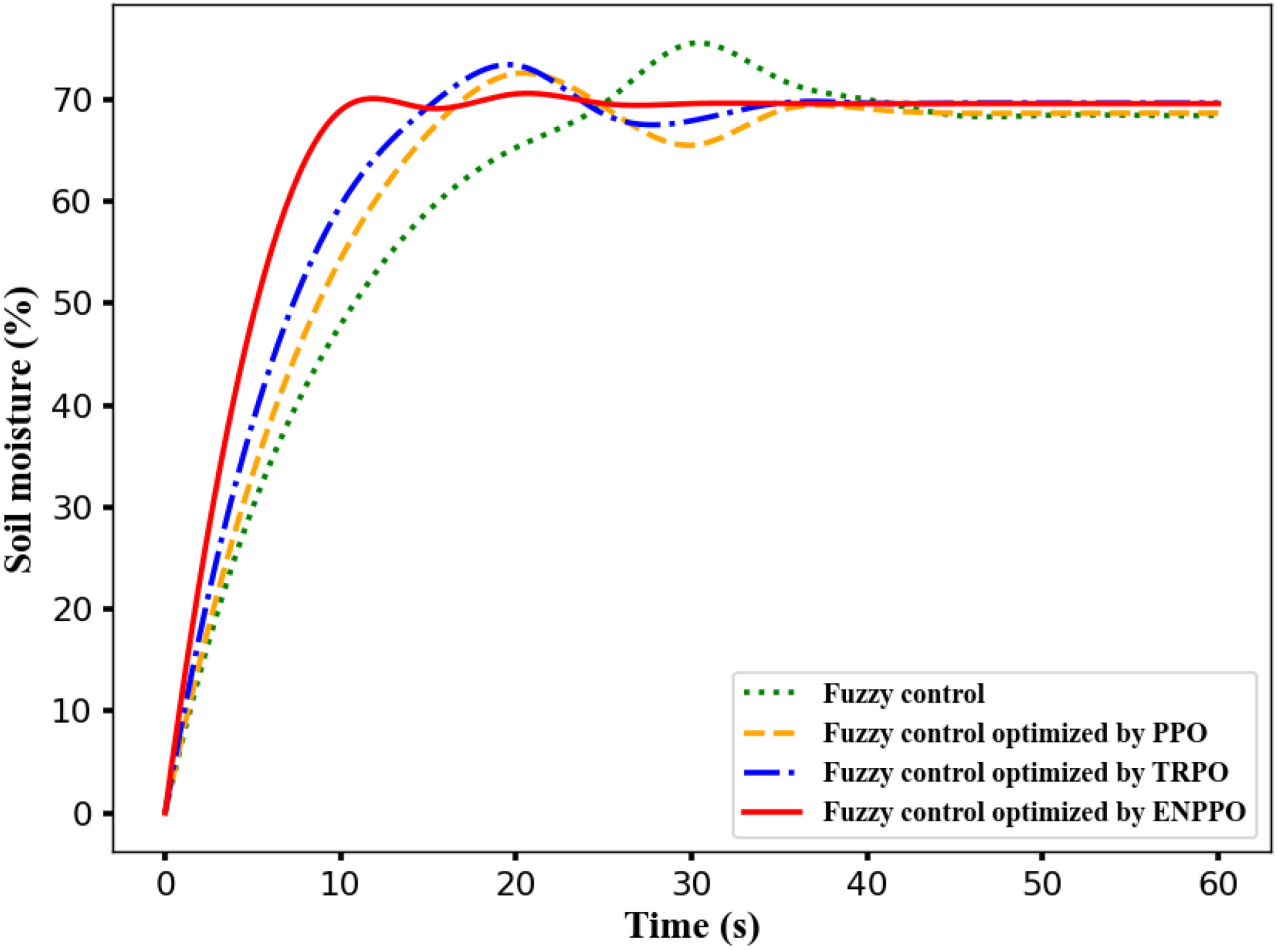
The iteration result of fuzzy control with different algorithms.

By optimizing the objective function introduced by the irrigation fuzzy controller (Jaiswal and Ballal, 2020), the optimal quantitative scaling factor parameters are obtained, and simulation experiments are carried out. **Figure 7** shows the iteration result of fuzzy control with different algorithms. The time for the fuzzy control system without any algorithm optimization to enter the stable state is 39.15s, and the average soil moisture value is 63.6%. The fuzzy controller optimized by the PPO, TRPO and ENPPO algorithm enters the steady state time is 8.94 s, 33.12s and 24.35s; its average soil moisture value is 64.53%, 66.25% and 68.67%. Compared with other three methods, the entering steady state time of ENPPO algorithm reduces 77.16%, 73.01% and 63.29%; its average soil moisture value is the closest to the expected value 70%. So, the fuzzy control optimized by the ENPPO algorithm has the shortest steady state time and the smallest overshoot (Ritchie et al., 2021).

The TRPO algorithm limits the update step size of the strategy through the trust region during the optimization process to avoid excessive changes in the strategy. However, it requires the calculation of second-order derivative information and a complex optimization process, which causes the low efficiency in high-dimensional state space. The PPO algorithm uses the “clip” function to limit the optimization strategy to the relatively small range, and the optimization efficiency is higher than the TRPO algorithm. However, the restrictions of the clip function are fixed and cannot be adjusted dynamically according to the state, which causes the PPO algorithm to have insufficient updates in complex dynamic environments, thereby prolonging the steady-state time of the fuzzy controller. The ENPPO algorithm introduces the negative incentive mechanism based on the PPO algorithm, which enhances the penalty for non-ideal states and speeds up the adjustment of the fuzzy controller to the steady-state region. It also dynamically adjusts the clip range, allowing the policy parameters to be flexibly updated according to the actual needs under different states. It avoids the problem of insufficient updates caused by the fixed clip range in the PPO algorithm, which improves the optimization efficiency and convergence performance of the ENPPO algorithm.

### 4.2 Robustness analysis

To verify the robustness of the proposed algorithm under different climatic conditions, this study conducted simulation experiments in the dry and wet seasons and compared the performance of PPO, TRPO, and ENPPO. The evaluation indicators included average soil moisture error, drainage event frequency(Indicates the number of drainage events that occurred in 100 hours), conductivity drift, water use efficiency (WUE, Indicates water use efficiency, usually the ratio of yield to water use), and the number of steps to converge to a stable state. **Table 1** shows the results of each indicator. Compared with PPO and TRPO, ENPPO reduced average soil moisture errors by 33.92%, 28.27%, 37.41% and 34.85% in the dry and wet seasons. It also reduced drainage event frequency by 57.59%, 51.50%, 63.42%, and 56.29%. It also reduced conductivity drift by 54.68%, 51.16%, 56.48% and 50.00%. It also improved WUE by 14.20%, 10.78%, 21.09% and 17.88%. It also reduced the number of convergence steps by 46.85%, 40.70%, 47.60%, and 44.09%, respectively. These results demonstrate that ENPPO significantly mitigates over-irrigation risk, reduces salt accumulation, improves water use efficiency, and accelerates convergence in both dry and wet seasons, demonstrating its robustness across climate conditions.

**Table 1:** The robustness indicators of each algorithm.

Algorithm	Season	Moisture error(%)	Drainage frequency (Number of events/100 hours)	EC drift(dS/m)	WUE	Steps to threshold
PPO	Dry	5.72	1.91	1.39	1.62	222
	Wet	7.11	3.80	2.16	1.47	271
TRPO	Dry	5.27	1.67	1.29	1.67	199
	Wet	6.83	3.18	1.88	1.51	254
ENPPO	Dry	3.78	0.81	0.63	1.85	118
	Wet	4.45	1.39	0.94	1.78	142

**Figure 8:**
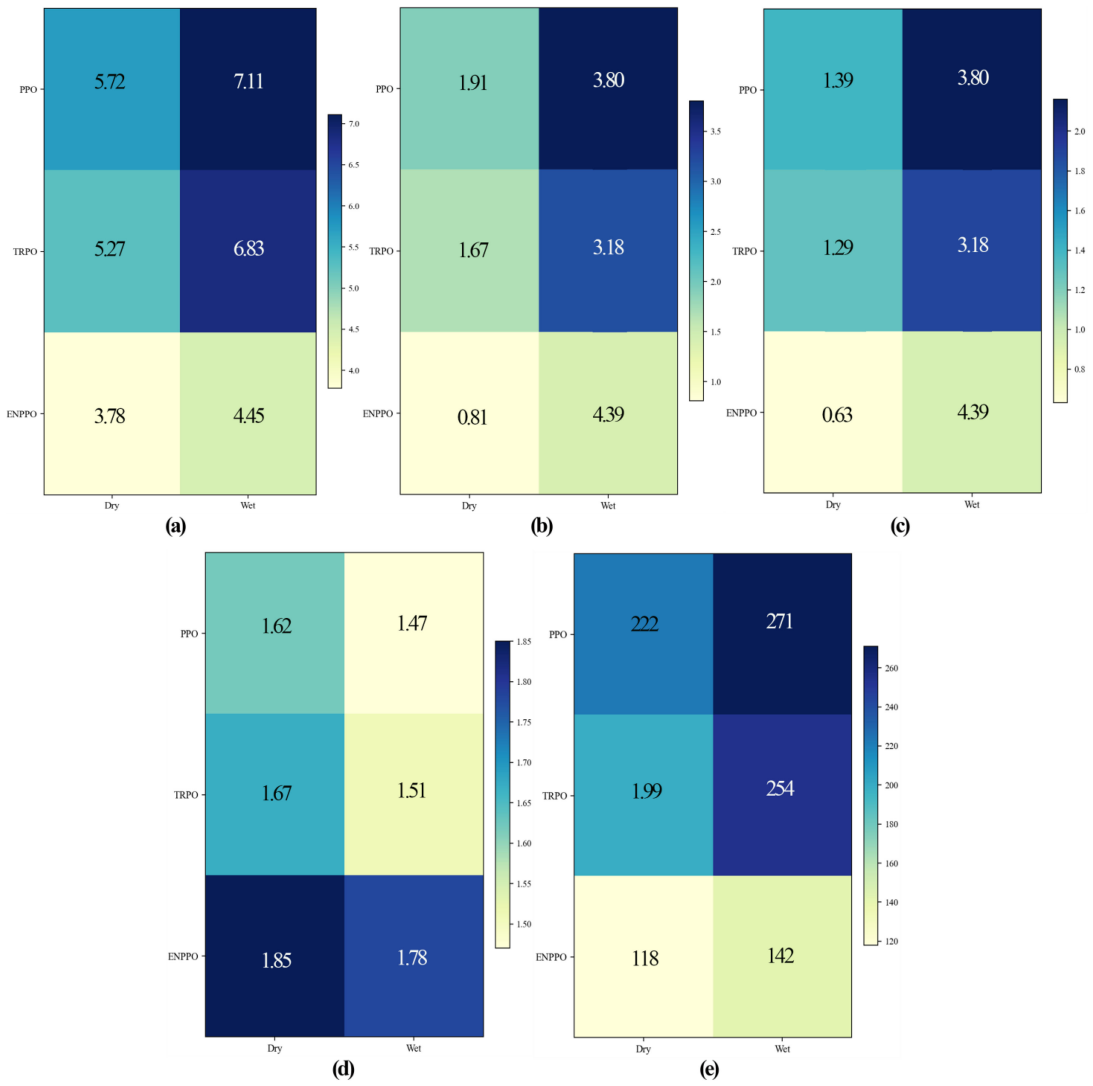
The robustness indicator matrix of each algorithm: *(a)* Moisture error (%); **(b)** Drainage frequency (Number of events/100 hours); **(c)** EC drift (dS/m); **(d)** WUE; **(e)** Steps to threshold.

**Figure 8** shows the robustness indicator matrix of each algorithm. The PPO algorithm showed significant increases in error and drainage in a humid environment. The TRPO algorithm had moderate stability but low convergence efficiency. The ENPPO algorithm showed faster convergence and higher water efficiency in both climate conditions, demonstrating that dynamic pruning and negative incentive mechanisms effectively improved adaptability and robustness under different climates.

### 4.3 Ablation analysis

To clarify the contributions of two key mechanisms in ENPPO (dynamic clipping and negative incentives), this study conducted ablation experiments on four variants: baseline PPO, PPO with dynamic clipping, PPO with negative incentives, and a combination of the two, ENPPO (Full). Evaluation metrics included average soil moisture error, number of drainage events, conductivity drift, WUE, and number of convergence steps. **Table 2** shows the specific results for each algorithm. Compared with the PPO baseline, ENPPO reduces the average soil moisture error by 36.69%, drainage events by 58.68%, conductivity drift by 50.00%, WUE by 22.15%, and convergence steps by 46.95%. Compared with PPO+Dynamic clipping, the reductions are 20.71%, 45.16%, and 36.88%, respectively, while WUE is improved by 11.66%, and convergence steps are shortened by 31.53%. Compared with PPO+Negative excitation, the reductions are 17.79%, 38.34%, and 27.05%, while WUE is improved by 4.60%, and convergence steps are shortened by 23.20%. The results show that PPO converges slowly and lacks robustness in complex environments; dynamic pruning improves the update flexibility of the strategy under state fluctuations, thereby accelerating convergence; negative incentives effectively suppress the risks of drainage and salt accumulation by strengthening the penalty for bad irrigation behavior; ENPPO, which combines the two mechanisms, simultaneously achieves higher water use efficiency and shorter convergence time, proving that both designs are necessary for performance improvement and have significant synergistic gains. In order to ensure robustness, statistical tests were performed. ANOVA results indicated that the differences among algorithms across both dry and wet seasons were statistically significant (p < 0.05). Post-hoc Tukey tests further confirmed that ENPPO outperformed PPO and TRPO in terms of soil moisture error, drainage reduction, and WUE improvements. Moreover, comparative benchmarks against traditional control strategies (PID, fuzzy logic, MPC) demonstrated that ENPPO not only surpassed reinforcement learning baselines but also consistently achieved more stable soil moisture and reduced water usage under real field conditions.

**Table 2:** The ablation analysis results of each algorithm.

Variant	Moisture error(%)	Drainage frequency (Number of events/100 hours)	EC drift(dS/m)	WUE	Steps to threshold
PPO baseline	6.35	2.88	1.78	1.49	262
PPO + Dynamic clipping	5.07	2.17	1.41	1.63	203
PPO + Negative incentive	4.89	1.93	1.22	1.74	181
ENPPO	4.02	1.19	0.89	1.82	139

### 4.4 Validation against real data and traditional control strategies

In order to further validate the ENPPO algorithm, its performance was evaluated against actual field data collected from the Tam Dao greenhouse and benchmarked with traditional control strategies, including PID, fuzzy logic and model predictive control (MPC). The PID control was implemented using Ziegler–Nichols tuning based on observed soil moisture response, with the target set at an optimal moisture level of 25%. The fuzzy logic control adopted a conventional design with soil moisture deviation and its rate of change as inputs, and irrigation volume as the output. For MPC, a linear predictive model with a six-hour prediction horizon was employed, utilizing historical evapotranspiration and sensor measurements as predictors. The testing period lasted for 15 consecutive days during the crop maturity stage, with real data collected at 30-minute intervals serving as the ground truth for performance assessment.

**Table 3** shows the comparative results for the different methods, including the average soil moisture maintained, cumulative water usage, standard deviation and the deviation from target moisture levels. ANOVA was conducted to determine the statistical significance of differences among methods. The results indicated significant differences among these methods (p < 0.05). Post-hoc Tukey tests confirmed the ENPPO algorithm statistically outperformed PID, MPC, fuzzy logic and PPO control methods in water-saving performance and stability of soil moisture maintenance. The performance superiority of ENPPO was not only evident compared to reinforcement learning baselines (PPO and TRPO), but also against traditional control methods such as PID, fuzzy logic and MPC. The added comparative study using actual greenhouse data further strengthened the robustness and practical relevance of the findings. Additionally, the statistical measures (including the confidence intervals and significance testing) provided the solid quantitative validation, which overcomes the earlier limitations regarding statistical robustness.

**Table 3:** The benchmark results of various algorithms.

Method	Avg. soil moisture (%)	Water used (L/m²)	Deviation from target (%)	Standard deviation (%)
PID	24.6 ± 2.4	68.2	5.2	2.4
MPC	25.3 ± 1.9	63.5	3.8	1.9
Fuzzy Logic	25.0 ± 2.0	65.1	4.2	2
PPO	25.1 ± 1.5	60.3	3.5	1.5
ENPPO	25.1 ± 1.0	58.4	2.8	1

### 4.5 Generalization *analysis*

This study also selected several representative greenhouse leafy vegetables—Chinese Cabbage, Shanghai Green and Komatsuna for the experimental evaluation. The first objective was Chinese cabbage. In the seedling stage, its demand for water was relatively low. The ENPPO algorithm can maintain stable water resource utilization efficiency within the soil moisture range of 20%-30% by optimizing the irrigation amount. In the growth period, due to the rapid growth of leaf area, the water demand increased significantly. It showed higher irrigation accuracy within the soil moisture range of 30%-50%, which reduces the water resource waste by 15% compared with the PPO and TRPO algorithms. Next was Shanghai Green. The ENPPO algorithm had good adaptability to the irrigation needs of Shanghai green at various growth stages. Especially in the mature stage, it can avoid over-irrigation and the soil moisture is controlled within the range of 40%-60%, which is highly consistent with the actual growth needs. Compared with the PPO and TRPO algorithms. The ENPPO algorithm reduced the irrigation frequency of Shanghai green by 12%, which reduces the irrigation cost. Finally was Komatsuna. Komatsuna was sensitive to the environmental conditions, especially under high light intensity conditions, and the evapotranspiration increases significantly. The ENPPO algorithm achieved accurate prediction of evapotranspiration by dynamically adjusting light intensity parameters, which achieved the best irrigation effect within the soil moisture range of 40%-70%. In the comprehensive yield impact analysis of Komatsuna, it improved the yield stability and its single-plant yield increased by 8.3% and 11.6% compared with the PPO and TRPO algorithms. These results show the applicability and advantages of the ENPPO algorithm in different growth stages and environmental conditions. 

## 5 Discussion

This study has several limitations that inform real-world deployment and future work: (i) the negative-excitation and dynamic-clipping components of ENPPO improve policy quality but increase training and online inference load and energy consumption, raising requirements for edge compute and potentially elevating hardware and maintenance costs; (ii) the method relies on multi-modal sensing and periodic calibration, so sensor drift, temporary outages, or higher fractions of sub-LOD/LOQ readings can degrade stability and accuracy—masking and uncertainty tagging help, but resilience under extreme missingness remains limited; (iii) demonstrated benefits are concentrated within the 20–80% soil-moisture operating band and the crops/climates studied here, and generalization to more extreme soils, salinity regimes, irrigation rules, or larger multi-house/multi-block coordination scenarios requires additional cross-site validation; (iv) mandatory safety guards and fallback routines protect operations but may trade off some optimality; and (v) data distributions drift with seasons and management, necessitating a long-term plan for retraining and model versioning (including data governance, A/B validation, and release criteria) to mitigate performance decay over time. These constraints point to concrete next steps: lightweight/distilled variants for low-compute platforms, more robust missing-data handling with adaptive calibration, cross-domain transfer and federated learning, and stronger interpretability and safety/compliance engineering for operations (Farooqui et al., 2024).

Therefore, the future research can optimize the computational complexity of the algorithm to make it more applicable on the low-cost hardware. The theoretical underpinnings of the ENPPO algorithm lie in its adaptive modification of the PPO surrogate loss function. Unlike the fixed clipping strategies, the dynamically adjusted clipping bounds reflect the varying uncertainty in state-action transitions, which ensures more context-sensitive policy updates. Additionally, the incorporation of a negative incentive term encourages the exploration away from unproductive policy regions, which mitigates the risk of premature convergence. Comparative experiments against DDPG further underscore the robustness of ENPPO in managing noisy, high-dimensional input spaces, highlighting its superiority in both convergence stability and irrigation control precision. This study also focused on solving the three major problems in irrigation control and targeted solutions. The first problem was the low irrigation efficiency of traditional systems. This study built the reinforcement of learning environment, considered multi-dimensional dynamic environmental variables and vegetable growth stages and introduced the ENPPO algorithm and negative incentive mechanism to reduce insufficient or excessive irrigation. Through experiments at different growth stages, in the seedling stage, the ENPPO algorithm controlled the irrigation amount within the range of 20%-30% soil moisture, which reduces water waste by 15% compared with PPO and TRPO. In the growth and maturity stages, the ENPPO algorithm adjusted the irrigation amount according to the evapotranspiration and light intensity, which increased water resource utilization by 18%. The second problem was that most systems rely on the complex equipment. In the sensor design, this study selected moderately cost soil moisture sensors and light intensity sensors, which combined with the simple data acquisition equipment to reduce hardware costs. The reinforcement learning model reduced the reliance on high-precision data and ensured the applicability of the algorithm in low-resolution sensor data environments (Farooqui and Ritika, 2019).

In the experiment, the ENPPO algorithm by using medium and low cost sensors still showed good performance. Among them, the accuracy of the soil moisture sensor was ±5%, and the accuracy of the light intensity sensor was ±10%. Under different sensor accuracies, the yield prediction error of the ENPPO algorithm was always less than 5%. The third problem was that the algorithm design of the existing system was too complex. The ENPPO algorithm realized the automatic adjustment of the strategy through the dynamic clipping function, which simplified the complexity of parameter configuration. It integrated different environmental variables and irrigation parameters into one. Users need to input sensor data, and the system can automatically generate the optimal irrigation plan. By comparing user operation steps, the ENPPO algorithm simplified the complexity of the system. Users does not need the complex configuration, they only need to input basic environmental data, and the system automatically outputs irrigation plans. Experiments show that the user operation time is reduced by about 40%.

## 6 Conclusions

This study proposed a greenhouse vegetable irrigation prediction method based on an improved deep reinforcement learning algorithm. It took several common vegetables as the research object, setted the reinforcement learning environment according to the water demand characteristics and greenhouse environment and designed the feedback function to construct the reinforcement learning algorithm of the greenhouse vegetable irrigation. The PPO algorithm based on the negative excitation were introduced to solve the problems of local optimality and discrete prediction, which improved the prediction accuracy. Experimental results proved that its performance was superior to the other three methods in terms of irrigation volume prediction and algorithm stability. It showed that the ENPPO algorithm can combine various environmental factors of the greenhouse to achieve the intelligent control and provide a more comprehensive solution for vegetables. It also adjusted the amount of irrigation according to real-time needs, minimized the use of water resources and reduced the production costs. It also improved the soil environmental quality and promoted the sustainable agricultural development.

## Data Availability

The original contributions presented in the study are included in the article/supplementary material. Further inquiries can be directed to the corresponding author.
